# Cirsiliol alleviates diabetic cardiomyopathy by inhibiting oxidative stress and improving energy metabolism through the PPAR-α/AMPK pathway

**DOI:** 10.1038/s41598-025-32157-w

**Published:** 2025-12-11

**Authors:** Jing Tao, Siqi Liu, Yunzhi Ling, Hanlin Bai, Lei Liu, Ru Yu, Guangling Li

**Affiliations:** 1 Department of Anesthesiology, the First Affiliated Hospital of Bengbu Medical University, Bengbu, China; 2 Department of Anesthesiology, the Second Affiliated Hospital of Bengbu Medical University, Bengbu, China; 3 Department of Anesthesiology, Affiliated Hospital of Jiangnan University, 1800 Lihu Avenue, Wuxi, 214122 Jiangsu China

**Keywords:** Diabetic cardiomyopathy, Cirsiliol, PPAR-α/AMPK, Oxidative stress, Cell apoptosis, Inflammatory response, Endocrine system and metabolic diseases, Medical research

## Abstract

Diabetic cardiomyopathy (DCM) is a core cause of heart failure in diabetic patients, with major pathological features including myocardial energy metabolism disorders, mitochondrial dysfunction, oxidative stress, and inflammatory cascades. This study investigates the mechanism by which the flavonoid compound Cirsiliol improves DCM by regulating the peroxisome proliferator-Activated receptor α (PPAR-α)/AMP-activated protein kinase (AMPK) signaling pathway. Using a high-glucose-treated H9C2 myocardial cell model and a streptozotocin-induced diabetic mouse model, the results show that Cirsiliol can dose-dependently increase myocardial cell survival, inhibit high-glucose-induced apoptosis, and significantly improve cardiac function in diabetic mice. Mechanistic studies indicate that Cirsiliol activates the PPAR-α/AMPK pathway, upregulates the expression of key fatty acid oxidation enzymes carnitine palmitoyltransferase 1 (CPT1) and p-acetyl-CoA carboxylase (ACC), restores mitochondrial membrane potential, reduces lipid peroxidation product malondialdehyde (MDA) levels, enhances superoxide dismutase activity, and inhibits the release of inflammatory factors such as Interleukin 6 (IL-6) and Tumor Necrosis Factor α (TNF-α). This study elucidates that Cirsiliol intervenes in energy metabolism imbalance, oxidative stress, and inflammatory responses through multiple targets, providing a new strategy for the treatment of DCM.

**Supplementary Information:**

The online version contains supplementary material available at 10.1038/s41598-025-32157-w.

## Introduction

Diabetic cardiomyopathy (DCM) is a significant cause of heart failure in diabetic patients, with core features including myocardial energy metabolism disorders, mitochondrial dysfunction, oxidative stress, and chronic inflammatory responses^1^. Epidemiological data show that diabetes is associated with pronounced heart failure and poorer clinical outcomes^2^. Studies have indicated that diabetic patients are at twice the risk of developing heart problems compared to non-diabetic populations, and cardiovascular complications are the leading cause of death in this group^3^. Although traditional anti-hyperglycemic drugs, such as metformin, can improve systemic metabolism by activating AMP-activated protein kinase (AMPK), their direct regulatory effect on myocardial mitochondrial homeostasis and the inflammatory microenvironment is limited^4^. Additionally, commonly used β-blockers, though they alleviate excessive sympathetic nerve activation, may further inhibit AMPK activity and exacerbate myocardial energy metabolism imbalance^5^. Therefore, targeting the regulation of AMPK and its synergistic signaling networks, such as peroxisome proliferator-activated receptor α (PPAR-α), while intervening in the oxidative stress-inflammation cascade, has become a new strategy for treating DCM.

AMPK, as a central sensor of cellular energy metabolism, promotes fatty acid oxidation through phosphorylation of acetyl-CoA carboxylase (ACC)^6^ and inhibits pathological myocardial hypertrophy mediated by mTORC1^7^. Recent studies have shown that AMPK can also alleviate mitochondrial fission and myocardial cell apoptosis by phosphorylating β-arrestin-1 to block excessive activation of the β-adrenergic receptor (β-AR)^5^. Notably, AMPK has a close interaction with the PPAR-α pathway: AMPK activation enhances the transcriptional activity of PPAR-α and upregulates the expression of key fatty acid oxidation enzymes such as carnitine palmitoyltransferase 1 (CPT1), thereby working synergistically to maintain myocardial energy homeostasis^8^. However, under high-glucose stress, the activity of the PPAR-α/AMPK signaling axis is significantly suppressed, leading to fatty acid metabolism disorders and the accumulation of reactive oxygen species (ROS), ultimately triggering myocardial cell apoptosis and ventricular remodeling^9^. This mechanism suggests that simultaneously activating the PPAR-α/AMPK pathway may provide a multi-target intervention approach to reverse the pathological progression of DCM.

Flavonoid compounds have gained significant attention in the treatment of metabolic diseases due to their antioxidant and anti-inflammatory properties. Cirsiliol (5,3’,4’-trihydroxy-6,7-dimethoxyflavone) is a natural product isolated from the Artemisia genus, which has shown significant biological activity in various research fields in recent years. In terms of anti-inflammatory effects, Cirsiliol effectively regulates IL-6-induced inflammatory responses by inhibiting JAK2 phosphorylation and the STAT3 signaling pathway, providing a potential strategy for the treatment of Interleukin 6 (IL-6)-related diseases^10^. In the anti-tumor field, Cirsiliol exhibits inhibitory effects in various tumor models, including oral cancer, esophageal squamous carcinoma, osteosarcoma, and colon cancer, by suppressing cell proliferation, inducing apoptosis (such as upregulating Bax, downregulating Bcl-2 and p53), and regulating the PI3K/Akt/NF-κB and STAT3 pathways^11–15^. Additionally, Cirsiliol significantly enhances solubility and in vivo anti-tumor effects by forming β-cyclodextrin inclusion complexes^16^. In neuroprotection, Cirsiliol can inhibit Aβ aggregation and reduce its cytotoxicity, providing new directions for the treatment of neurodegenerative diseases^17^. Research has also discovered its inhibitory effect on bacterial ATP synthase, suggesting its potential as an antibiotic adjuvant^18^. Pharmacological mechanism studies further reveal the correlation between its antioxidant activity and the catechol structure^19^. These multifaceted research advancements lay a crucial foundation for the clinical application of Cirsiliol. However, its regulatory effects on myocardial energy metabolism and inflammatory responses in DCM remain unclear. Therefore, this study proposes the scientific hypothesis that Cirsiliol improves DCM cardiac injury by synergistically activating the PPAR-α/AMPK pathway to inhibit high-glucose-induced mitochondrial membrane potential loss, Bax/Bcl-2-mediated apoptosis, and IL-6/MCP-1-driven inflammation.

This study systematically evaluates the intervention effects of Cirsiliol on myocardial cell survival, cardiac function, and metabolic disorders using high-glucose-treated H9C2 myocardial cells and streptozotocin (STZ)-induced diabetic mouse models. Furthermore, it investigates the molecular mechanism by which Cirsiliol regulates oxidative stress, apoptosis, and inflammation networks through the PPAR-α/AMPK-CPT1 axis. The research findings for the first time reveal the multidimensional cardioprotective effects of Cirsiliol, providing experimental evidence for the development of DCM treatment drugs targeting energy metabolism-oxidative stress cross pathways.

## Materials and methods

### Materials

H9C2 rat cardiomyocytes (Procell, Catalog # CL-0089) were cultured in DMEM (Gibco, Catalog # 11965092) supplemented with 10% fetal bovine serum (Gibco, Catalog # A5256701) and 1% penicillin-streptomycin (Biyuntian, Catalog # C0222). Streptozotocin (Sigma-Aldrich, Catalog # S0130) was dissolved in 0.1 M citrate buffer (pH 4.5). Cirsiliol (MedChemExpress, Catalog # HY-110399) was prepared in dimethyl sulfoxide (DMSO) (MedChemExpress, Catalog # HY-Y0320C), with a final concentration less than 0.1% in the cell culture medium. Antibodies for Bax (Catalog # 5023), Bcl-2 (Catalog # 3498), SOD1 (Catalog # 37385), SOD2 (Catalog # 13141), IL-6 (Catalog # 12153), MCP-1 (Catalog # 2029), PPAR-α (Catalog # 2435), AMPK (Catalog # 5831), LKB1 (Catalog # 3047), and ACADM (Catalog # 4321) were purchased from Cell Signaling Technology. The antibody for IL-8 (Catalog # ab289967) was purchased from Abcam. Horseradish peroxidase (HRP)-conjugated goat anti-rabbit secondary antibody (Catalog # A0208) and goat anti-mouse secondary antibody (Catalog # A0216) were purchased from Biyuntian Biotechnology Co., Ltd.

### Animal and cell models

#### Diabetic cell model

H9C2 cells and primary cardiomyocytes were incubated in high-glucose DMEM medium (35 mM glucose) for 48 h to simulate hyperglycemic damage. In the drug intervention experiment, Cirsiliol was divided into low-dose group (20 µM) and high-dose group (50 µM) according to the effective concentration range from preliminary experiments^20^. The specific groups are as follows:

1. Normal Control Group: Cells were cultured in DMEM medium with standard glucose concentration (5.5 mM glucose);
2. High Glucose Model Group (HG Group): Cells were exposed to 35 mM glucose in DMEM medium for 48 h;
3. High Glucose + Cirsiliol Low Dose Group (HG + 20 µM Group): Before high glucose treatment, cells were pretreated with 20 µM Cirsiliol for 24 h;
4. High Glucose + Cirsiliol High Dose Group (HG + 50 µM Group): The pretreatment method was the same as the low-dose group, but the Cirsiliol concentration was 50 µM.

### Diabetic mouse model

Male C57BL/6 mice (8 weeks old, weighing 18–22 g, specific pathogen-free) were purchased from Beijing Vital River Laboratory Animal Technology Co., Ltd. Before the experiment, all mice were housed in an SPF-grade environment, with a controlled temperature of 24 ± 1 °C, humidity of 55% ± 5%, and a 12-hour light-dark cycle, with free access to food and water. After one week of adaptive feeding, the mice were used for the experiment.

The diabetic model was established using the STZ induction method. STZ was dissolved in freshly prepared 0.1 mol/L citrate buffer (pH 4.5), mixed thoroughly under an ice bath in the dark to ensure the drug’s activity. The mice were randomly divided into two groups: the normal control group (Normal, *n* = 10) and the diabetic model group (Diabetic, *n* = 20). Mice in the model group received a daily intraperitoneal injection of STZ (50 mg/kg/day) for 5 consecutive days, with a 6-hour fasting period before injection to enhance drug sensitivity. Mice in the normal control group were injected with an equal volume of citrate buffer.

72 h after the last injection, fasting blood glucose levels were measured using a Roche Accu-Chek blood glucose meter (after 6 h of fasting). Mice with blood glucose levels ≥ 11.1 mmol/L (200 mg/dL) were considered to have successfully developed diabetes. Cirsiliol (purity ≥ 98%) was suspended in physiological saline (containing 5% DMSO and 5% Tween-80) to prepare a 2 mg/mL suspension. The diabetic mice were further randomly divided into two groups:

- Diabetic group (*n* = 10): continued regular feeding without drug intervention;
- Diabetic-Cirsiliol group (*n* = 10): orally administered 20 mg/kg Cirsiliol daily for 4 weeks^21^.

The normal group received no drug intervention and only basic physiological parameters were monitored. During the experiment, mouse body weight, food intake, and water consumption were recorded daily, and fasting blood glucose was checked weekly. All procedures were conducted in accordance with the AAALAC international standards for laboratory animal care and were approved by the Institutional Animal Care and Use Committee of Bengbu Medical University. It is noteworthy that within 24 h after the STZ injection, all mice were supplemented with a 5% glucose solution to prevent acute hypoglycemic shock. This measure significantly reduced the animal mortality rate during the model construction process. This study was approved by the Ethics Committee of Hubei University of Medicine and confirm that all methods were performed in accordance with ARRIVE guidelines and other relevant guidelines and regulations.

### Cell viability

Cell viability was assessed using the CCK-8 method. H9C2 cardiomyocytes were seeded at a density of 1 × 10^4^ cells per well in a 96-well plate, with PBS buffer filling the outer wells to minimize the edge effect interference. The experiment was divided into two treatment systems:

- Concentration Gradient Experiment: Cells were exposed to Cirsiliol solutions at concentrations of 20, 40, 60, 80, and 100 µM for 24 h to assess the drug’s toxicity through a dose-response relationship.
- Time Gradient Experiment: Six treatment groups were set up: normal medium group (Normal), low-dose group (20 µM), high-dose group (50 µM), and corresponding high glucose environment groups (HG medium). Samples were collected at 12, 24, 36, 48, 60, and 72 h post-treatment.

After treatment, 10 µL of CCK-8 reagent (Biosharp #BS350A) was added to each well, with careful addition along the wall to avoid bubbles. The plates were incubated in a 37 °C incubator for 2 h in the dark. During incubation, the color change was observed every 30 min to ensure the OD value remained within the linear detection range of 1.0–2.0. Absorbance was measured using a Thermo Scientific Varioskan LUX microplate reader with dual-wavelength detection (primary wavelength 450 nm, reference wavelength 650 nm) to eliminate light scattering interference caused by the high-glucose medium. Experimental data were normalized to the untreated group as the baseline for analysis.

### Cell apoptosis

Cells (approximately 1 × 10^6^ cells/mL) were collected and centrifuged at 500–1000 rpm for 5 min to remove the culture medium. They were washed twice with pre-cooled PBS to remove residual components. The Annexin V-FITC/PI double staining method (BD Biosciences, catalog #556547) was used. After digestion of adherent cells with trypsin without EDTA, cells were resuspended in 1×Binding Buffer (containing 10 mM HEPES/NaOH, 140 mM NaCl, 2.5 mM CaCl₂, pH 7.4) at a concentration of 1–3 × 10⁶ cells/mL. 100 µL of the cell suspension was added to a flow cytometry tube, and 5 µL of Annexin V-FITC (final concentration 1 µg/mL) and 5 µL of PI (final concentration 50 µg/mL) were sequentially added. The cells were gently mixed and incubated in the dark for 15 min at room temperature. After staining, 400 µL of pre-cooled Binding Buffer was added to terminate the reaction, and the samples were immediately analyzed using a flow cytometer. Flow cytometry was performed using the BD FACSCanto™ II system, with 488 nm laser excitation and fluorescence detection at 530/30 nm (FITC) and 610/20 nm (PI). Data were analyzed using quadrant gating: Annexin V⁻/PI⁻ represents live cells (lower-left quadrant), Annexin V⁺/PI⁻ represents early apoptotic cells (lower-right quadrant), and Annexin V⁺/PI⁺ represents late apoptotic/necrotic cells (upper-right quadrant). Single-stain controls were used to calibrate fluorescence compensation, and debris was excluded using FSC/SSC parameters.

### Western blotting

Total protein was extracted from cells or heart tissues using RIPA lysis buffer (containing 1% protease and phosphatase inhibitors, Biyuntian, catalog #P0013B). Protein concentration was determined using the BCA method (Biyuntian, catalog #P0010). Equal amounts of protein (30 µg) were separated using 10% SDS-PAGE gel, and then transferred onto polyvinylidene fluoride (PVDF) membranes (Millipore, catalog #IPVH00010). The membranes were blocked with 5% non-fat milk for 1 h and incubated overnight at 4 °C with primary antibodies (dilution 1:1000). Afterward, membranes were incubated with HRP-conjugated secondary antibodies (dilution 1:5000) at room temperature for 1 h. Enhanced chemiluminescence (ECL; Bio-Rad, catalog #1705061) was used to develop the protein bands, and ImageJ software (NIH) was used for quantitative analysis.

### Enzyme-linked immunosorbent assay (ELISA)

Heart tissues were homogenized in PBS (1:10 weight/volume ratio), and the homogenate was centrifuged at 12,000 ×g for 15 min at 4 °C. According to the manufacturer’s instructions, commercial ELISA kits (Elabscience, catalog #E-EL-M0044 / E-EL-M3063) were used to measure the levels of IL-6 and TNF-α in the supernatant. Absorbance was measured at 450 nm, and cytokine concentrations were calculated based on standard curves.

### Malondialdehyde (MDA)

MDA levels in heart tissues were quantified using the Thiobarbituric Acid Reactive Substances (TBARS) method. Fresh heart tissue samples were washed with physiological saline and then homogenized in pre-cooled homogenization buffer to prepare a 10% tissue homogenate. After centrifugation at 12,000 ×g for 15 min at 4 °C, the supernatant was collected for analysis. According to the Biyuntian TBARS assay kit (catalog #S0131) instructions, tissue homogenate was mixed with thiobarbituric acid (TBA) reaction solution in a 1:2 volume ratio and incubated at 95 °C for 30 min. After the reaction was stopped with an ice bath, the reaction mixture was centrifuged, and the supernatant was collected. Absorbance was measured at 532 nm using a spectrophotometer, and MDA levels were calculated using a standard MDA curve.

### Superoxide dismutase (SOD) activity

The SOD activity was measured using the WST-8 method (Biyuntian, catalog #S0101). The experimental system included superoxide anions catalyzed by xanthine oxidase (XOD) reacting with WST-8 to produce a water-soluble formazan product. Heart tissue homogenate supernatant was mixed with the reaction mixture containing 0.2 mM xanthine, 0.1 mM EDTA, 0.05 mM WST-8, and 0.01 U/mL XOD, and incubated at 37 °C in the dark for 30 min. Absorbance at 450 nm was measured using a microplate reader. The SOD activity was calculated using the formula, defined as the enzyme amount required to inhibit 50% of the superoxide radical-mediated reduction reaction, and the results were normalized by the protein content (measured by BCA assay).

### Mitochondrial membrane potential

Mitochondrial membrane potential was assessed using the JC-1 fluorescent probe method (Biyuntian, catalog #S2006). Fresh mouse heart tissue was minced and digested with type II collagenase (0.1–0.2 mg/mL) at 37 °C for 20–30 min. After digestion was terminated, the cells were filtered through a 100 μm mesh and centrifuged (300 g, 5 min) to obtain a single-cell suspension. Cells were then stained with 5 µg/mL JC-1 staining solution (containing 1×JC-1 buffer) at 37 °C for 30 min in the dark. After staining, cells were washed three times with pre-warmed JC-1 washing buffer (300 g, 5 min) to remove unbound probes. Fluorescence imaging was performed using an Olympus IX73 fluorescence microscope with excitation/emission wavelengths of 514/529 nm (green monomer fluorescence) and 585/590 nm (red aggregate fluorescence). The ratio of the average intensity of red to green fluorescence in randomly selected fields was quantified using ImageJ software. A lower red/green fluorescence ratio indicates mitochondrial depolarization and early apoptosis. A positive control group treated with CCCP (carbonyl cyanide m-chlorophenyl hydrazone) was included to ensure the effectiveness of the staining system.

### Hematoxylin and eosin (H&E) staining

Heart tissue was fixed in 4% paraformaldehyde for 24 h, paraffin-embedded, and sectioned into 4 μm thick slices. H&E staining was performed to assess inflammatory cell infiltration and structural changes. Images were captured using a Nikon Eclipse E100 microscope.

### Echocardiography

Cardiac function was assessed using the Vevo 2100 ultrasound system (Fujifilm VisualSonics) under 1.5% isoflurane anesthesia. Left ventricular parameters, including left ventricular ejection fraction (LVEF), left ventricular end-diastolic volume (LVEDV), left ventricular end-systolic volume (LVESV), and fractional shortening (LVFS), were obtained from M-mode images. Data from three consecutive cardiac cycles were analyzed for each mouse.

### Statistical analysis

In this study, data were presented as mean ± standard error of the mean (SEM). All experiments were performed with at least three independent replicates, as detailed in the figure legends. Statistical comparisons were made using appropriate tests, including one-way or two-way analysis of variance (ANOVA) with Tukey’s post-hoc test, as specified in the results section. Statistical significance was considered at *p* < 0.05.

## Results

### Dose- and time-dependent protective effects of cirsiliol in high-glucose-induced myocardial cell injury model

**Fig. 1:**
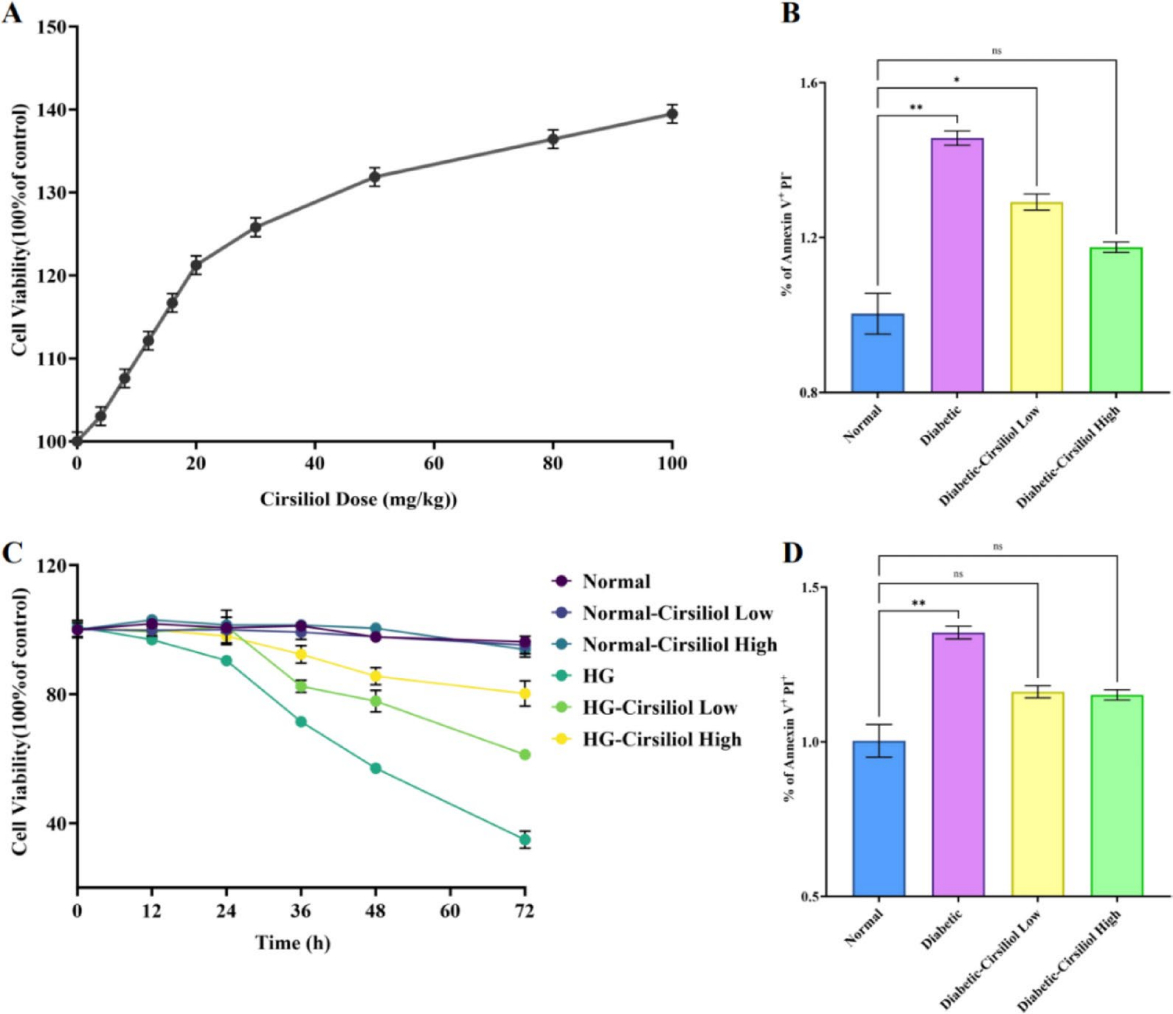
Dose-dependent protective effects of Cirsiliol on high-glucose-induced diabetic cardiomyocyte viability and apoptosis. The experiment shows the results of treating H9C2 cardiomyocyte cell lines with high glucose to simulate diabetic cardiovascular damage and induce a diabetic cell model. In the experiment, (**A**) cell viability was assessed using the CCK-8 assay after treatment with different doses of Cirsiliol; (**B**) cell viability was measured by Annexin V + PI- staining; (**C**) cell viability was analyzed at different time points in various treatment groups; (**D**) apoptosis rates were determined by Annexin V + PI + staining. The results for all assays are presented as mean ± SEM. Each experiment was repeated at least three times to ensure reproducibility. Statistical significance is indicated as follows: *p < 0.05, **p < 0.01, and ns for no significance.

This study established a diabetic cardiovascular injury model by treating H9C2 myocardial cells with high glucose, systematically evaluating the intervention effects of Cirsiliol on high glucose-induced decreases in cell viability and apoptosis. As shown in Fig. 1A, Cirsiliol significantly increased cell viability in a dose-dependent manner: within the range of 0–60 mg/kg, the viability increased from the baseline of 100% to 130% with increasing dosage; when the dosage reached 60–100 mg/kg, the effect plateaued, and the viability stabilized at 130–140%, indicating that the maximum cell protective effect of the compound could be achieved at doses above 60 mg/kg. Annexin V/PI double-staining analysis (Fig. 1B and D) revealed that high glucose treatment significantly increased the proportion of late apoptotic/necrotic cells (*p* < 0.01) and reduced the survival rate of early apoptotic cells (*p* < 0.01). After Cirsiliol intervention, the survival rate of early apoptotic cells was significantly increased (*p* < 0.05), and the proportion of late apoptotic/necrotic cells was significantly reduced (*p* < 0.05), suggesting that Cirsiliol exerts its protective effects by inhibiting the apoptotic pathway. Time-gradient experiments (Fig. 1C) showed that the cell viability of the high glucose (HG) group decreased to 50% at 48 h and further deteriorated at 72 h, while the low-dose (Low) and high-dose (High) Cirsiliol intervention groups maintained cell viability at 80% and 60%, respectively, at 72 h. Notably, Cirsiliol treatment alone in the normal group had no significant effect on cell viability (ns), indicating that its protective effect is specific to high glucose-induced damage. The above results suggest that Cirsiliol effectively alleviates high glucose-induced decreases in myocardial cell viability and apoptosis through a dose- and time-dependent mechanism.

### The improvement effects of cirsiliol on metabolic disorders and cardiac dysfunction in STZ-Induced diabetic mice

**Fig. 2:**
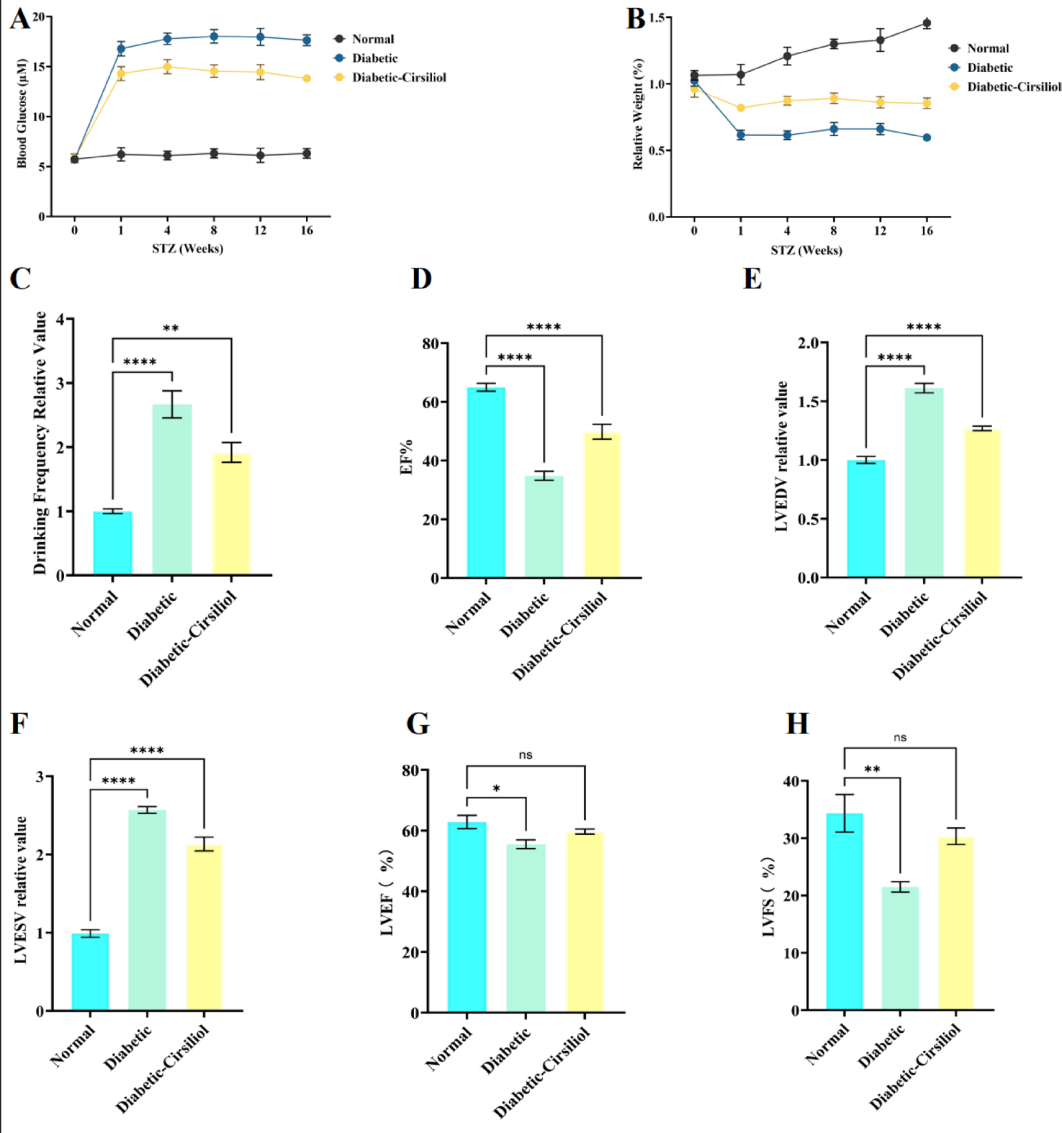
Evaluation of the effects of Cirsiliol on metabolic disorders and cardiac dysfunction in streptozotocin-induced diabetic mice. The results of behavioral and physiological assessments in three groups of mice using a streptozotocin (STZ)-induced diabetic mouse model are presented. The three groups include the control group, the diabetic group (Diabetic group), and the diabetic-Cirsiliol-treated group (20 mg/kg, Diabetic-Cirsiliol group). The experimental content includes: (**A**) Regular monitoring of blood glucose levels in mice to ensure the establishment of the diabetic model; (**B**) Regular monitoring of body weight in diabetic and control mice to ensure the establishment of the diabetic model; (**C**) Recording of food and water intake, i.e., regular monitoring of food and water consumption in diabetic and control mice; (**D**) Left ventricular end-diastolic volume (LVEDV); (**E**) Left ventricular end-systolic volume (LVESV); (**F**) Left ventricular ejection fraction (LVEF); (**G**) Left ventricular fractional shortening (LVFS). The results for all assays are presented as mean ± SEM. Each experiment was repeated at least three times to ensure reproducibility. Statistical significance is indicated as follows: *p < 0.05, **p < 0.01, ****p < 0.0001, and ns for no significance.

This study used a streptozotocin (STZ)-induced diabetic mouse model to evaluate the intervention effects of Cirsiliol (20 mg/kg) on metabolic disorders and cardiac dysfunction. The experimental results showed that the blood glucose level in the diabetic group was significantly elevated by approximately three times compared to the normal group (Fig. 2A, *p* < 0.01), whereas the blood glucose level in the Cirsiliol treatment group was reduced by about 20% compared to the diabetic group, but still higher than the normal group (*p* < 0.01). The body weight of diabetic mice decreased by approximately 40% (Fig. 2B, *p* < 0.01), and the drinking frequency increased by about 2.7 times due to hyperglycemia (Fig. 2C, *p* < 0.0001). After Cirsiliol treatment, the body weight partially recovered, and the drinking frequency decreased (*p* < 0.01), indicating that it effectively alleviates diabetes-related metabolic abnormalities.

In terms of cardiac function, the left ventricular ejection fraction (LVEF) in the diabetic group decreased compared to the normal group (Fig. 2D, *p* < 0.0001), while the left ventricular end-diastolic volume (LVEDV) and end-systolic volume (LVESV) increased (Fig. 2E and F, *p* < 0.0001), and the left ventricular fractional shortening (LVFS) significantly decreased (Fig. 2G and H, *p* < 0.01), suggesting impaired ventricular dilation and systolic function. After Cirsiliol treatment, the LVEF recovered to 70% of the normal group (*p* < 0.05), the LVEDV and LVESV decreased (*p* < 0.0001), and the LVFS increased compared to the diabetic group (*p* < 0.05). The above results indicate that Cirsiliol significantly alleviates cardiac dysfunction in diabetic mice by improving metabolic disorders and reversing ventricular remodeling.

### Cirsiliol inhibits the expression of inflammatory factors to alleviate cardiac damage in DCM

**Fig. 3:**
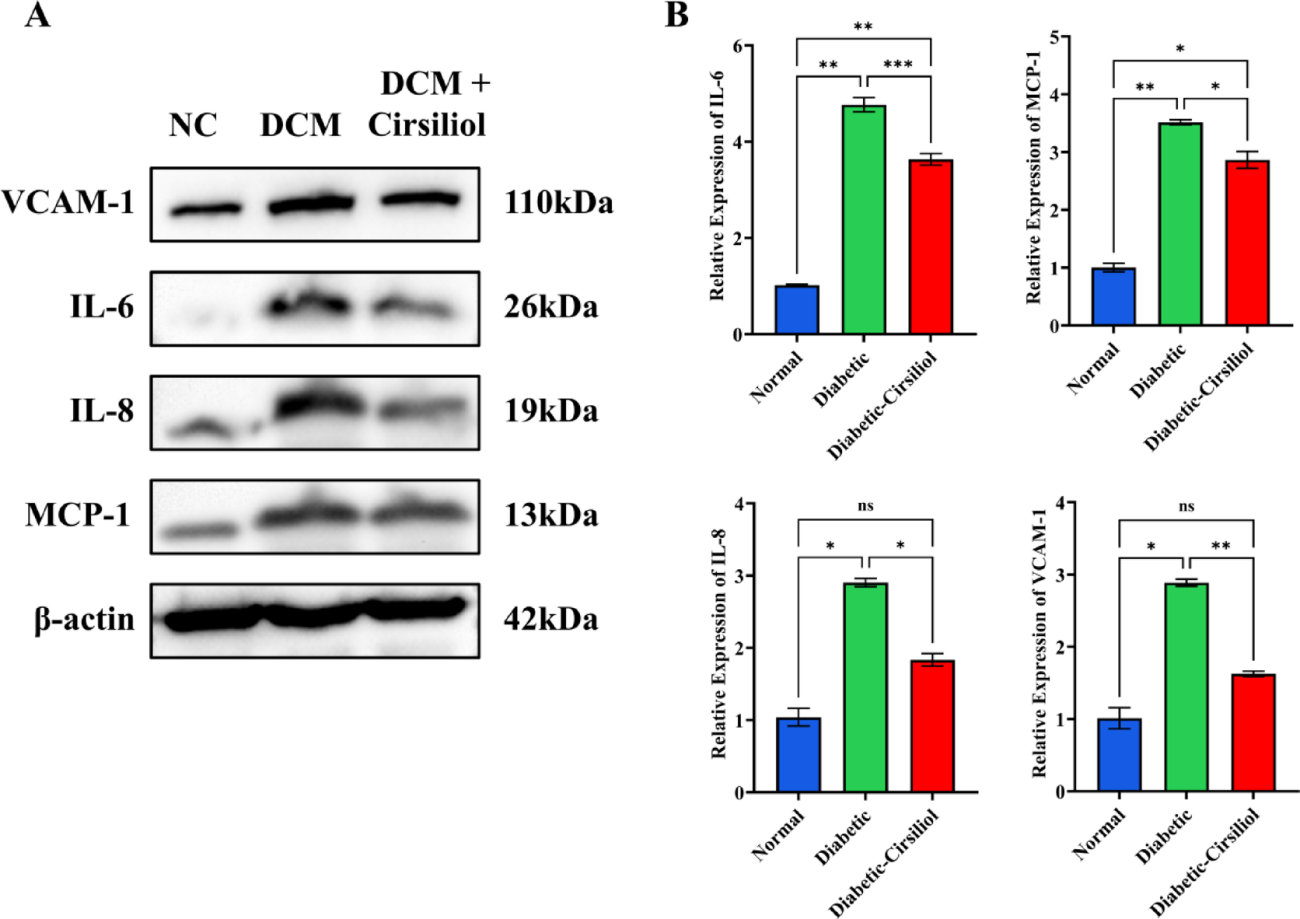
Expression levels of inflammation-related proteins in cardiac tissues of different treatment groups. (**A**,**B**) The results of the detection of inflammatory factor levels in cardiac tissues, which were assessed using Western Blot (WB) in the experiment. The results for all assays are presented as mean ± SEM. Each experiment was repeated at least three times to ensure reproducibility. Statistical significance is indicated as follows: **p < 0.01, ***p < 0.001, and ****p < 0.0001.

This study measured the expression levels of inflammatory factors IL-6, IL-8, MCP-1, and VCAM-1 in the heart tissue of DCM mice and evaluated the intervention effects of Cirsiliol (20 mg/kg) (Fig. 3). The experimental results showed that compared to the normal group, the expression levels of IL-6, IL-8, MCP-1, and VCAM-1 were significantly elevated in the heart tissue of the DCM group (Fig. 3A, *p* < 0.0001). Specifically, the expression of IL-6 and MCP-1 increased approximately 3 times (Fig. 3B and C), while IL-8 and VCAM-1 were elevated by about 2.8 times (Fig. 3D and E), indicating that the cardiac inflammatory response was significantly activated in the DCM model.

After Cirsiliol treatment, the expression levels of these inflammatory factors in the heart tissue of DCM mice were significantly reduced. The expression levels of IL-6 and MCP-1 returned to 3.5 times and 2.8 times that of the normal group, while the expression levels of IL-8 and VCAM-1 decreased to 1.9 times and 1.6 times those of the normal group, respectively. These results suggest that Cirsiliol exerts an anti-inflammatory effect by inhibiting the release of inflammatory factors. In conclusion, Cirsiliol significantly reduces the expression levels of IL-6, IL-8, MCP-1, and VCAM-1 in the heart tissue of DCM mice, effectively alleviating the cardiac inflammatory response.

### Cirsiliol alleviates cardiac damage in DCM by regulating oxidative stress, cell apoptosis, and inflammatory markers

**Fig. 4:**
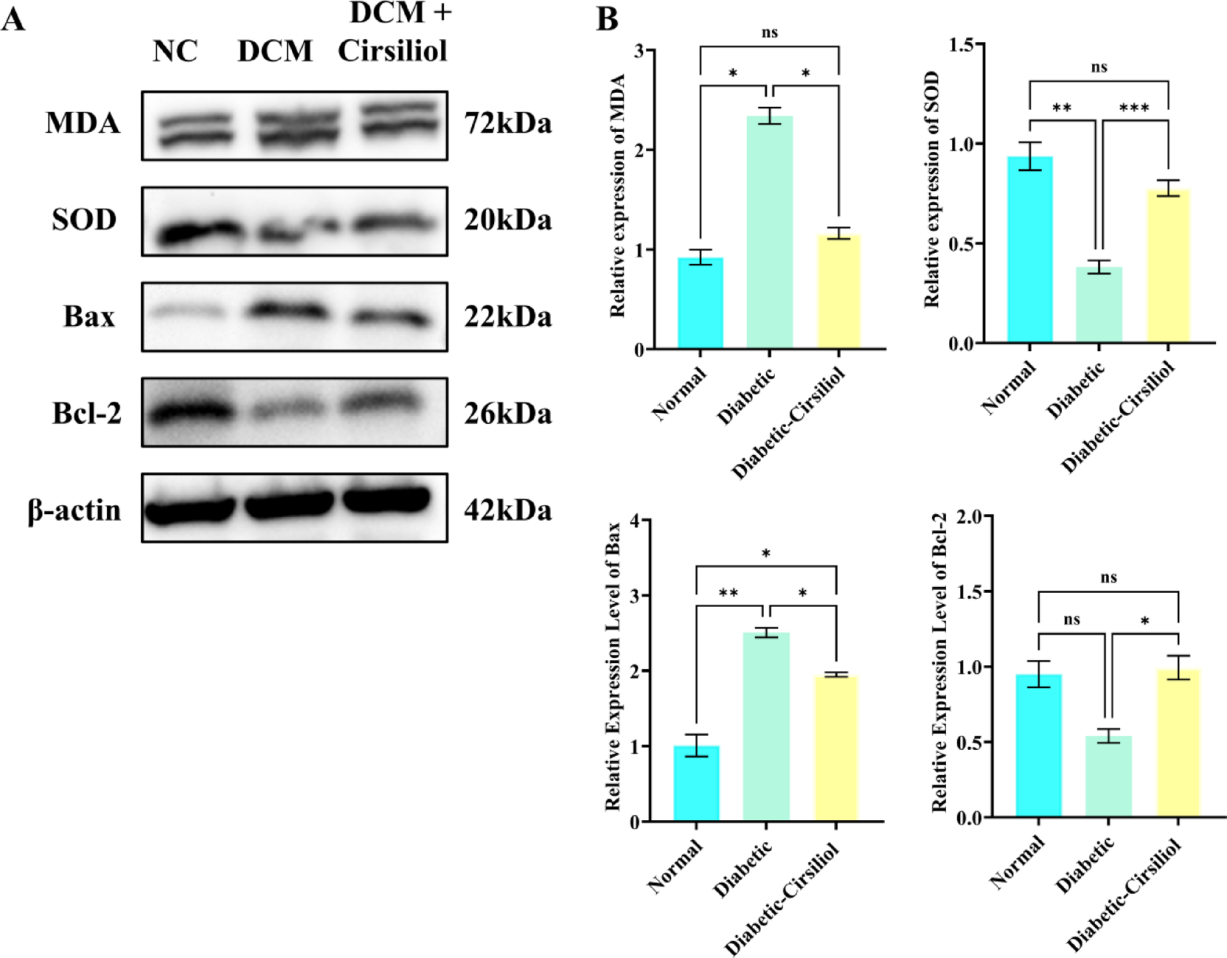
Expression levels of oxidative stress, apoptosis-related proteins, and inflammatory markers in cardiac tissues of different treatment groups. (**A**,**B**) The expression of markers related to oxidative stress, apoptosis responses in cardiac tissues was detected using Western Blot (WB) methods and quantitative analysis. The results for all assays are presented as mean ± SEM. Each experiment was repeated at least three times to ensure reproducibility. Statistical significance is indicated as follows: **p < 0.01, ***p < 0.001, ****p < 0.0001, and ns for no significance.

This study analyzed the expression levels of oxidative stress, apoptosis, and inflammation-related markers in the heart tissue of DCM mice using Western Blot and enzyme-linked immunosorbent assay (ELISA) (Fig. 4). The results showed that, compared to the normal group, the level of lipid peroxidation product MDA in the heart tissue of the DCM group was significantly increased (Fig. 4B, *p* < 0.0001), while the expression of the antioxidant enzyme SOD was significantly decreased (Fig. 4C, *p* < 0.01). After Cirsiliol intervention, MDA levels were significantly reduced (*p* < 0.001) compared to the DCM group, and SOD levels were increased (*p* < 0.01), suggesting that its protective effect in DCM might be through reducing lipid peroxidation, restoring SOD activity, and alleviating oxidative stress damage.

In terms of apoptosis-related indicators, the expression of the pro-apoptotic protein Bax was significantly higher in the DCM group compared to the normal group (Fig. 4D, *p* < 0.0001), while the anti-apoptotic protein Bcl-2 expression was significantly reduced (Fig. 4E, *p* < 0.01), leading to an increase in the Bax/Bcl-2 ratio. After Cirsiliol treatment, Bax expression was significantly reduced (Fig. 4D, *p* < 0.01), and Bcl-2 expression increased (*p* < 0.05), suggesting that it reduces myocardial cell apoptosis by inhibiting the pro-apoptotic pathway. Furthermore, ELISA detection showed that the expression levels of the inflammatory factors IL-6 (Fig. 4F, *p* < 0.0001) and TNF-α (Fig. 4G, *p* < 0.0001) were significantly elevated in the DCM group, confirming the anti-inflammatory effect of Cirsiliol. These results indicate that Cirsiliol alleviates cardiac damage in DCM through multiple pathways: by reducing MDA levels, restoring SOD activity to alleviate oxidative stress, inhibiting Bax-mediated apoptosis, and downregulating the expression of IL-6/TNF-α to relieve inflammation. This provides a basis for targeted intervention in diabetic cardiovascular complications.

### Inhibition of the PPAR-α/AMPK pathway in DCM and the partial repair effect of cirsiliol

**Fig. 5:**
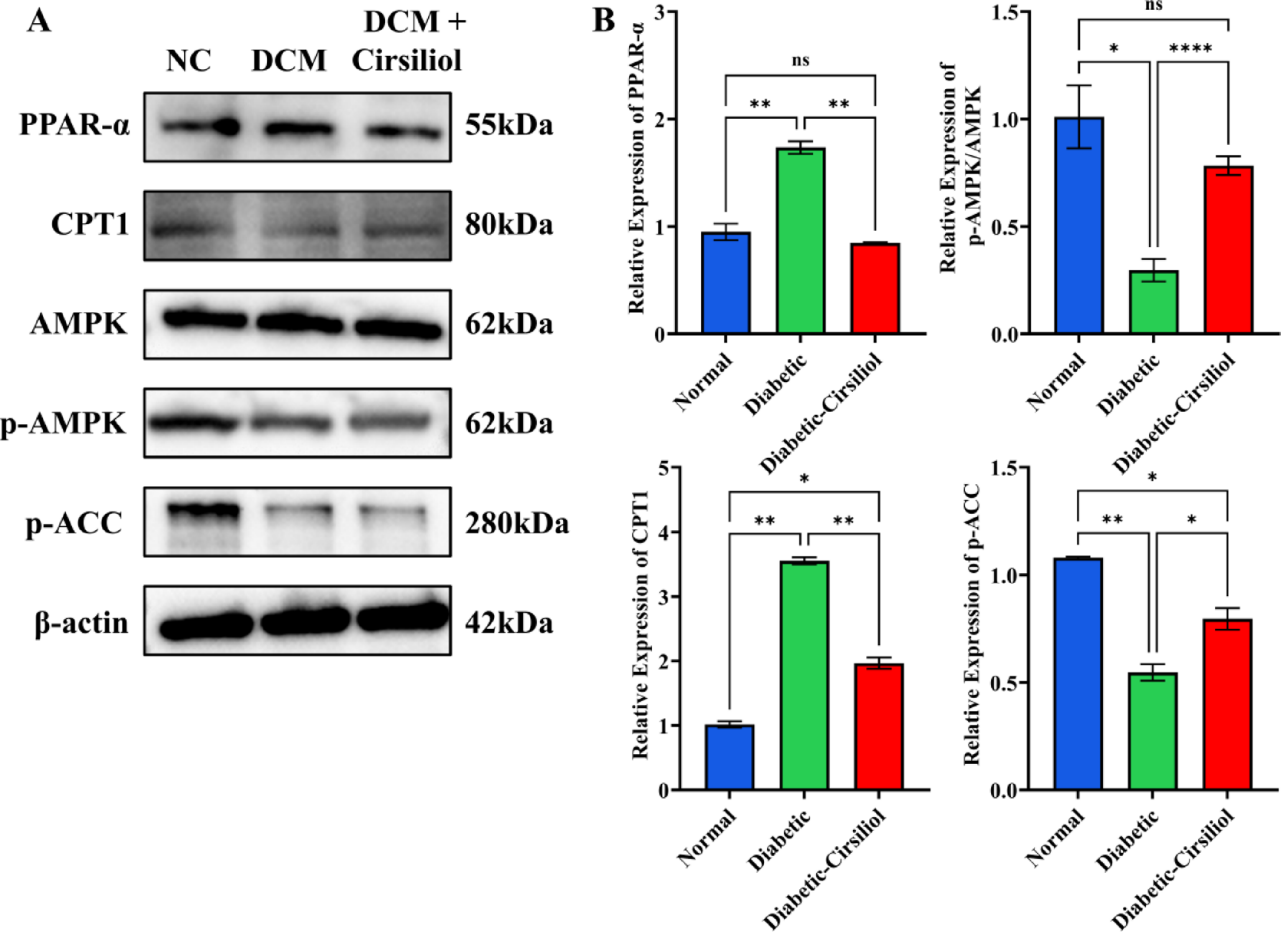
Cirsiliol regulates cardiomyocyte metabolism in diabetic mice via the PPAR-α/AMPK pathway. (**A**,**B**) The expression of proteins related to the PPAR-α/AMPK pathway was detected using Western Blot (WB). The results for all assays are presented as mean ± SEM. Each experiment was repeated at least three times to ensure reproducibility. Statistical significance is indicated as follows: **p < 0.01, ***p < 0.001, and ns for no significance.

This study examined the expression levels of PPAR-α/AMPK pathway-related proteins in the heart tissue of DCM mice (Fig. 5). The results showed that, compared to the normal group, the expression levels of PPAR-α (*p* < 0.01), p-AMPK (*p* < 0.01), and its downstream target CPT1 (*p* < 0.001) were significantly decreased in the heart tissue of DCM mice (Fig. 5B-D), indicating that the PPAR-α/AMPK pathway activity is significantly suppressed in the diabetic state. Additionally, the expression level of p-ACC, a key enzyme in fatty acid synthesis, was also significantly decreased in the DCM group (Fig. 5E, *p* < 0.001), suggesting fatty acid metabolism dysfunction in DCM.

After treatment with Cirsiliol (20 mg/kg), the expression levels of PPAR-α, p-AMPK, CPT1, and p-ACC in the heart tissue of the mice were partially restored, but did not reach the normal group levels (Fig. 5B-D, *p* < 0.01). This suggests that its repair effect may be mediated through the activation of the PPAR-α/AMPK signaling axis, promoting fatty acid oxidation to improve the metabolic abnormalities in DCM. The above findings indicate that the suppression of PPAR-α/AMPK pathway activity leads to fatty acid metabolic disorders in DCM, and Cirsiliol improves metabolic imbalance by partially restoring the expression of PPAR-α/AMPK and CPT1, providing a basis for targeted modulation of energy metabolism pathways to alleviate diabetic cardiac injury.

### Functional validation of PPAR-α in mediating the effects of cirsiliol

**Fig. 6:**
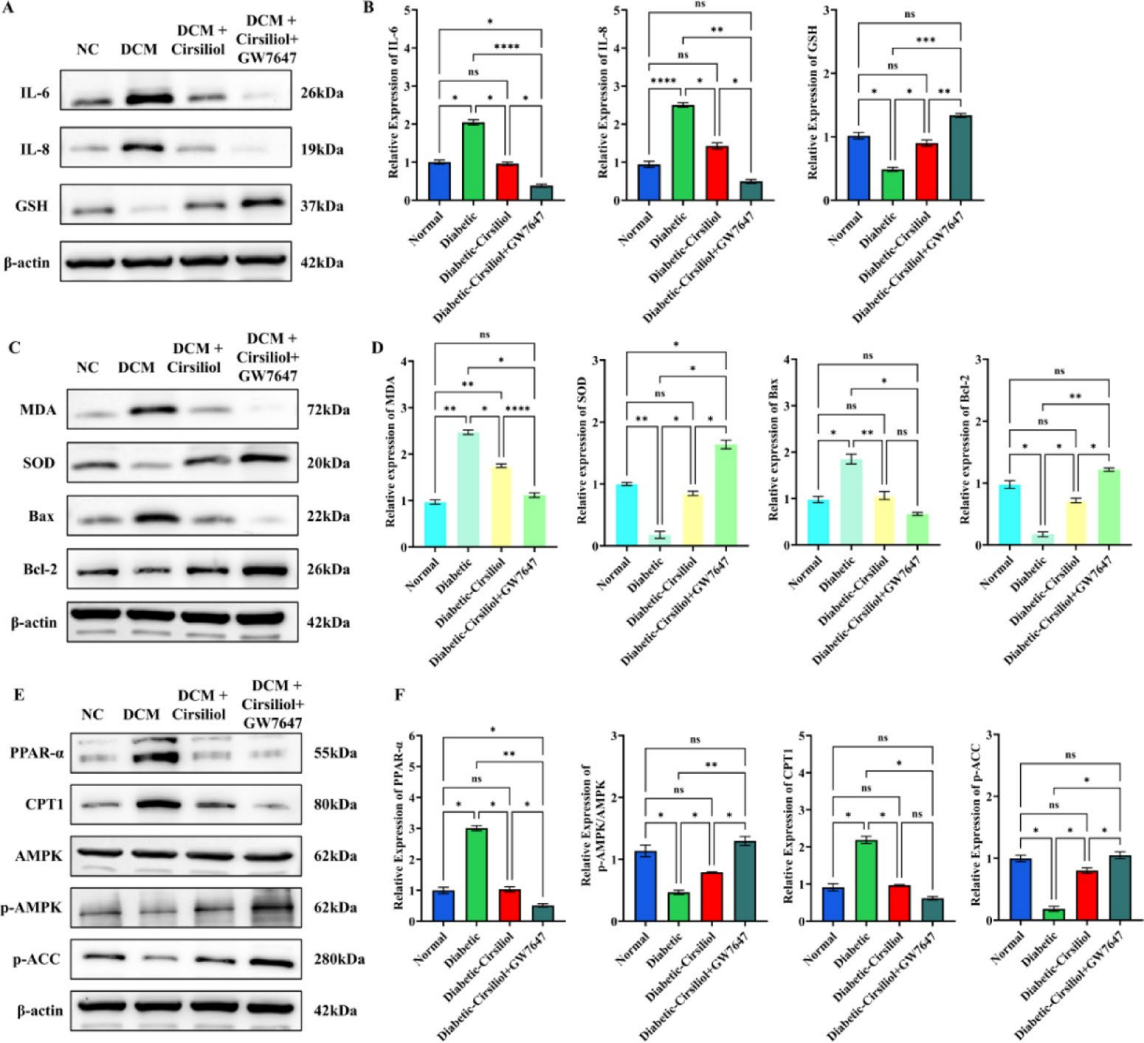
PPAR-α agonist GW7647 enhances the antioxidative, anti-inflammatory, and metabolic regulatory effects of Cirsiliol in diabetic cardiomyopathy. (**A**,**B**) Representative Western blots and quantitative analyses of inflammatory cytokines (IL-6, IL-1β) and antioxidant marker GSH in tissues. (**C**,**D**) Western blot results and quantification of oxidative stress and apoptosis-related proteins, including MDA, SOD, Bax, and Bcl-2. (**E**,**F**) Western blot analysis of PPAR-α, CPT1, AMPK, phosphorylated AMPK (p-AMPK), and phosphorylated ACC (p-ACC). The results for all assays are presented as mean ± SEM. Each experiment was repeated at least three times to ensure reproducibility. Statistical significance is indicated as follows: **p < 0.01, ***p < 0.001, and ns for no significance.

With the PPAR-α agonist GW7647, Cirsiliol’s suppression of IL-6 and IL-1β was further strengthened, and GSH levels increased (Fig. 6A,B). GW7647 also enhanced resistance to oxidative stress and apoptosis, as indicated by lower MDA, elevated SOD, reduced Bax, and increased Bcl-2 (Fig. 6C,D). In addition, GW7647 amplified Cirsiliol-induced activation of the PPAR-α/AMPK metabolic axis, increasing PPAR-α, CPT1, p-AMPK, and p-ACC expression (Fig. 6E,F).

**Fig. 7:**
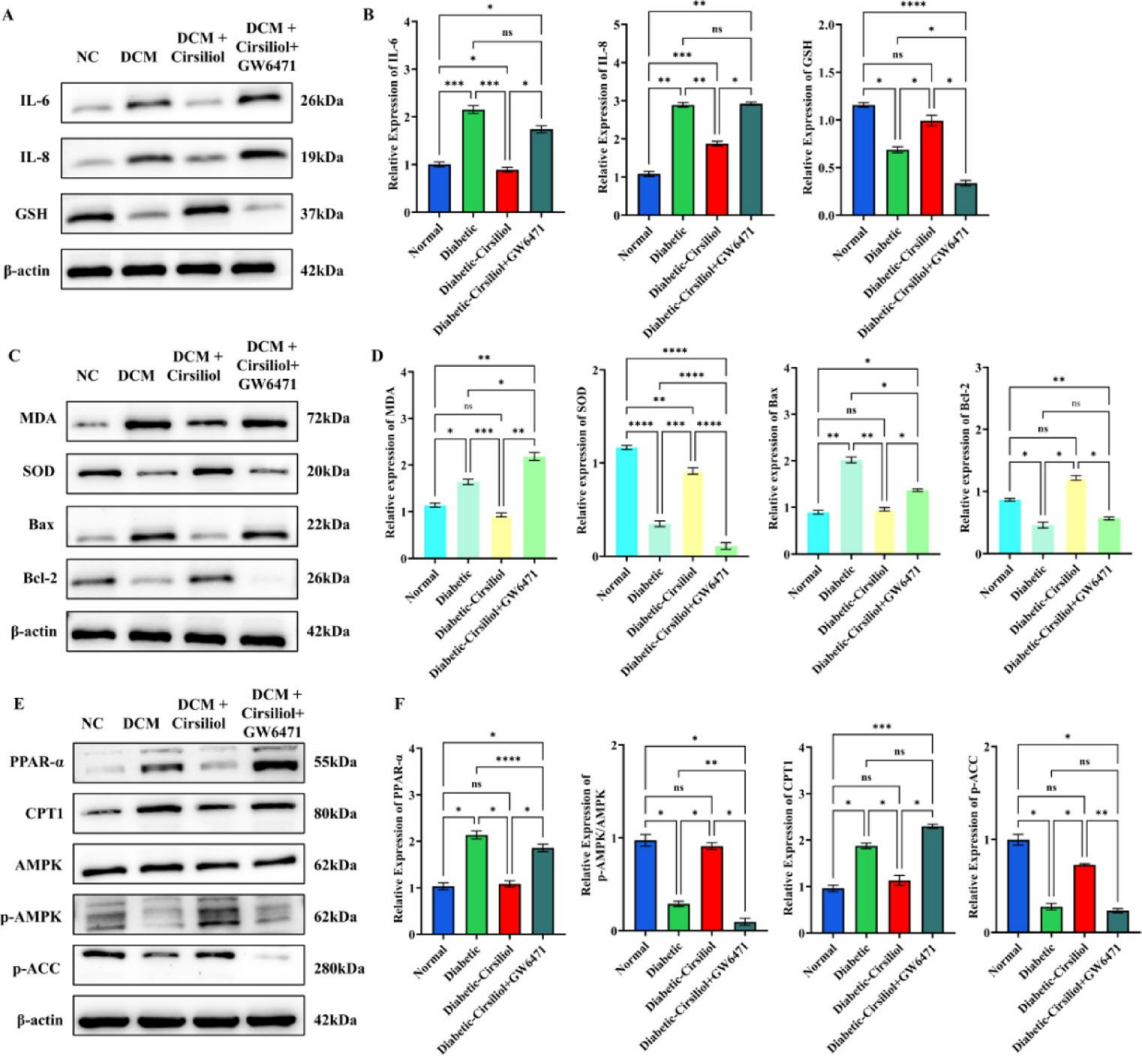
PPAR-α inhibition by GW6471 abolishes the metabolic benefits of Cirsiliol. (**A**,**B**) Western blot analysis and quantitative analysis of inflammatory cytokines (IL-6, IL-1β) and GSH levels. (**C**,**D**) Protein expression of oxidative stress and apoptosis-related markers, including MDA, SOD, Bax, and Bcl-2. (**E**,**F**) Western blots and quantification of metabolic regulators (PPAR-α, CPT1) and energy-sensing pathway markers (AMPK, p-AMPK, p-ACC). The results for all assays are presented as mean ± SEM. Each experiment was repeated at least three times to ensure reproducibility. Statistical significance is indicated as follows: **p < 0.01, ***p < 0.001, and ns for no significance.

In contrast, PPAR-α inhibition by GW6471 attenuated nearly all beneficial effects of Cirsiliol. IL-6 and IL-1β levels rebounded, and GSH declined (Fig. 7A,B). GW6471 also reversed Cirsiliol’s reductions in MDA and its increases in SOD, Bax/Bcl-2 balance returning toward a pro-apoptotic state (Fig. 7C,D). Metabolically, GW6471 suppressed PPAR-α and CPT1 expression and reduced AMPK and ACC phosphorylation (Fig. 7E,F), indicating impaired fatty-acid oxidation. These findings confirm that Cirsiliol’s anti-inflammatory, antioxidative, anti-apoptotic, and metabolic benefits are strongly dependent on PPAR-α signaling.

## Discussion

This study systematically reveals, for the first time, the protective effect of the flavonoid compound Cirsiliol in DCM and its molecular mechanisms. By integrating in vitro high glucose-induced cardiomyocyte injury models with STZ-induced diabetic mouse models, we confirmed that Cirsiliol significantly improves ventricular systolic function, reduces ventricular dilation, inhibits cardiomyocyte apoptosis (decrease in Bax/Bcl-2 ratio), and alleviates oxidative stress (SOD, MDA) and inflammatory response. Mechanistic studies indicate that Cirsiliol exerts its effects by synergistically activating the PPAR-α/AMPK signaling axis, regulating the expression of key fatty acid oxidation molecules CPT1 and p-ACC, restoring mitochondrial membrane potential, and inhibiting the release of pro-inflammatory factors, thereby providing multidimensional intervention for diabetic cardiac injury.

Long-term hyperglycemia can lead to myocardial metabolic disorders, microvascular damage, and oxidative stress, which in turn trigger cardiomyocyte apoptosis, fibrosis, and heart dysfunction. This may ultimately progress to heart failure, arrhythmia, or even sudden death^22^. However, meta-analysis shows that intensive glucose control, compared to conventional glucose control, does not significantly reduce the risk of hospitalization due to heart failure^23^. To date, no effective methods have been established for treating myocardial lipotoxicity. The core mechanisms of the disease involve multiple pathological interactions such as imbalance of glucose and lipid metabolism, accumulation of reactive oxygen species (ROS), excessive release of inflammatory factors, and abnormal activation of the renin-angiotensin system (RAS)^24^. Our study found that the protective effect of Cirsiliol is closely related to its multilayered inhibition of the oxidative stress-apoptosis-inflammation cascade. In a high-glucose environment, the decreased expression of SOD1/SOD2 leads to ROS accumulation, whereas Cirsiliol directly antagonizes oxidative stress damage by restoring SOD activity and significantly reducing lipid peroxidation marker MDA levels. Meanwhile, Cirsiliol significantly inhibits the expression of pro-apoptotic protein Bax and upregulates the expression of anti-apoptotic protein Bcl-2, reversing the activation of the apoptotic pathway, thus maintaining cardiomyocyte survival. Previous studies have shown^13^ that Cirsiliol regulates osteosarcoma cell apoptosis in a concentration-dependent manner, which may be significantly related to the intrinsic apoptotic pathway, leading to mitochondrial-related apoptosis. Notably, we found that Cirsiliol not only affects the restoration of mitochondrial membrane potential, but also significantly inhibits the excessive release of inflammatory factors such as IL-6, IL-8, MCP-1, and VCAM-1 in diabetic hearts. These inflammatory factors can exacerbate myocardial fibrosis and dysfunction^25,26^. This multidimensional regulatory action suggests that Cirsiliol is not limited to a single molecular target but rather breaks the vicious cycle of apoptosis-inflammation driven by oxidative stress, achieving comprehensive intervention for myocardial injury.

Excessive lipid deposition and mitochondrial dysfunction are key pathological features in the development and progression of DCM. Studies have shown that PPAR-α, as a central regulator of lipid metabolism, enhances the process of fatty acid β-oxidation through transcriptional regulation of downstream target genes, effectively alleviating cardiomyocyte lipotoxicity^27,28^. Additionally, AMPK, as an intracellular energy sensor, dynamically regulates fatty acid metabolism to maintain mitochondrial energy homeostasis^29^. Further mechanistic exploration revealed that Cirsiliol activates the nuclear receptor PPAR-α and the energy sensor AMPK, leading to upregulation of the downstream effector molecule CPT1 and increased phosphorylation of ACC. As a rate-limiting enzyme in fatty acid β-oxidation, sustained activation of CPT1 effectively promotes the transport of long-chain fatty acids into mitochondria, while the decline in malonyl-CoA levels mediated by ACC further relieves the inhibitory effect on mitochondrial fatty acid uptake^30^, thereby alleviating the characteristic lipotoxic accumulation in DCM (Supplementary information).

Furthermore, recent studies have shown that defects in mitochondrial gene translation are closely associated with energy metabolism imbalance in cardiomyocytes. For example, AGO2 protein, by activating the translation process of mitochondrial genes Cytb and Nd4, enhances the efficiency of the electron transport chain and reduces reactive oxygen species (ROS) generation, thereby improving heart function^31^. In this study, we observed that Cirsiliol significantly restores mitochondrial membrane potential and promotes fatty acid oxidative metabolism, suggesting that it may optimize the energy metabolism network by regulating the AGO2-TUFM mitochondrial gene translation complex. Notably, SIRT3-mediated protein malonylation modulates the mitochondrial localization of AGO2, and downregulation of SIRT3 activity has been confirmed as a significant molecular feature in DCM. Whether Cirsiliol activates SIRT3 to promote AGO2 mitochondrial translocation and thereby cooperates with the PPAR-α/AMPK signaling network to improve mitochondrial function is a scientific question worth further investigation. This is a hypothesis that warrants future research. Overall, the findings suggest that the mechanisms through which Cirsiliol improves energy metabolism homeostasis are central to its cardiac protective effects.

In this study, we used the STZ-induced type 1 diabetic mouse model to investigate the cardioprotective effects of Cirsiliol. However, we acknowledge that this model is limited in its ability to fully replicate the complexities of human diabetic cardiomyopathy, which is more commonly associated with type 2 diabetes (T2D) and metabolic syndrome^32^. While the STZ model serves as a valid representation of T1D-associated DCM, it does not capture the insulin resistance, obesity, and metabolic disturbances typical of T2D patients. These differences may influence the efficacy of Cirsiliol in humans with T2D and metabolic syndrome, conditions that are often characterized by altered PPAR-α/AMPK signaling. In light of this, we suggested that the cardioprotective effects of Cirsiliol in T2D models, which better reflect the human condition, should be investigated in future studies. These studies should also consider the impact of metabolic syndrome, as insulin resistance and obesity may affect the activation of key pathways such as PPAR-α/AMPK. Furthermore, the potential for Cirsiliol to modify the progression of DCM in obese or insulin-resistant mice remains to be explored, as the pathway interactions may differ from those observed in STZ-induced models.

However, it is noted that the long-term safety of Cirsiliol administration and its pharmacokinetic properties were not evaluated in this study. Given its potential as a therapeutic agent, it is crucial to consider its bioavailability, metabolic stability, and toxicity profile. Preliminary studies suggest that Cirsiliol exhibits moderate bioavailability, which can be enhanced through formulation strategies such as cyclodextrin complexes^33^. Furthermore, while Cirsiliol has shown promising biological activities, including anti-inflammatory and antioxidant effects, its potential toxicity remains underexplored. Studies on similar flavonoids have shown low toxicity at therapeutic doses^20,34^, but further research on Cirsiliol’s pharmacokinetics, long-term administration, and any dose-dependent toxic effects are needed to fully assess its safety for clinical use. Additionally, Cirsiliol’s metabolic stability should be studied to determine its potential for drug-drug interactions. This would be especially important when considering combination therapies in patients with diabetes and cardiovascular diseases, who often require multiple medications. Although the current study provides compelling evidence of Cirsiliol’s potential efficacy in DCM, its safe use in humans must be thoroughly evaluated through long-term toxicity studies and clinical trials.

This study also has the following limitations: First, the experimental model is mainly based on STZ-induced type 1 diabetic mice, which may not fully reflect the pathophysiology of diabetic cardiomyopathy in humans, where type 2 diabetes and metabolic syndrome are more prevalent. Future studies should investigate the effects of Cirsiliol in insulin-resistant or obese models to better understand its potential in treating DCM in patients with type 2 diabetes or metabolic syndrome. Second, although we found that Cirsiliol can activate the PPAR-α/AMPK pathway, its direct targets and upstream regulatory mechanisms have not been fully clarified. Also we did not address the long-term safety of Cirsiliol administration or its pharmacokinetics. Future studies should investigate the bioavailability, metabolic stability, and toxicity profile of Cirsiliol in preclinical and clinical models. Finally, the study did not evaluate the long-term safety of Cirsiliol administration or its efficacy in large animal models, and further preclinical translational research is needed.

## Conclusion

This study reveals the multi-target protective mechanism of the flavonoid compound Cirsiliol in DCM. By synergistically activating the PPAR-α/AMPK signaling axis, Cirsiliol significantly enhances CPT1-mediated fatty acid oxidation. Meanwhile, it inhibits Bax/Bcl-2-dependent apoptotic pathways by restoring mitochondrial membrane potential. Additionally, this compound downregulates excessive release of inflammatory factors such as IL-6 and TNF-α, effectively blocking the pathological cascade of oxidative stress and inflammation. These findings not only highlight Cirsiliol’s multidimensional action network in mitigating myocardial injury by regulating energy metabolism homeostasis, apoptosis balance, and the inflammatory microenvironment but also provide a theoretical basis for the development of targeted therapeutic strategies for DCM.

## Supplementary Information

Below is the link to the electronic supplementary material.

Supplementary Material 1

## Data Availability

The data that support the findings of this study are available from the corresponding author, Guangling Li, upon reasonable request. This study was approved by the Ethics Committee of Hubei University of Medicine and confirm that all methods were performed in accordance with ARRIVE guidelines and other relevant guidelines and regulations.
