# Supplementary figures and images for: Cirsiliol alleviates diabetic cardiomyopathy by inhibiting oxidative stress and improving energy metabolism through the PPAR-α/AMPK pathway
Source: Sci Rep. 2025 Dec 11;16:2331. doi: 10.1038/s41598-025-32157-w (PMC12816094; doi:10.1038/s41598-025-32157-w)

**Figure 3**


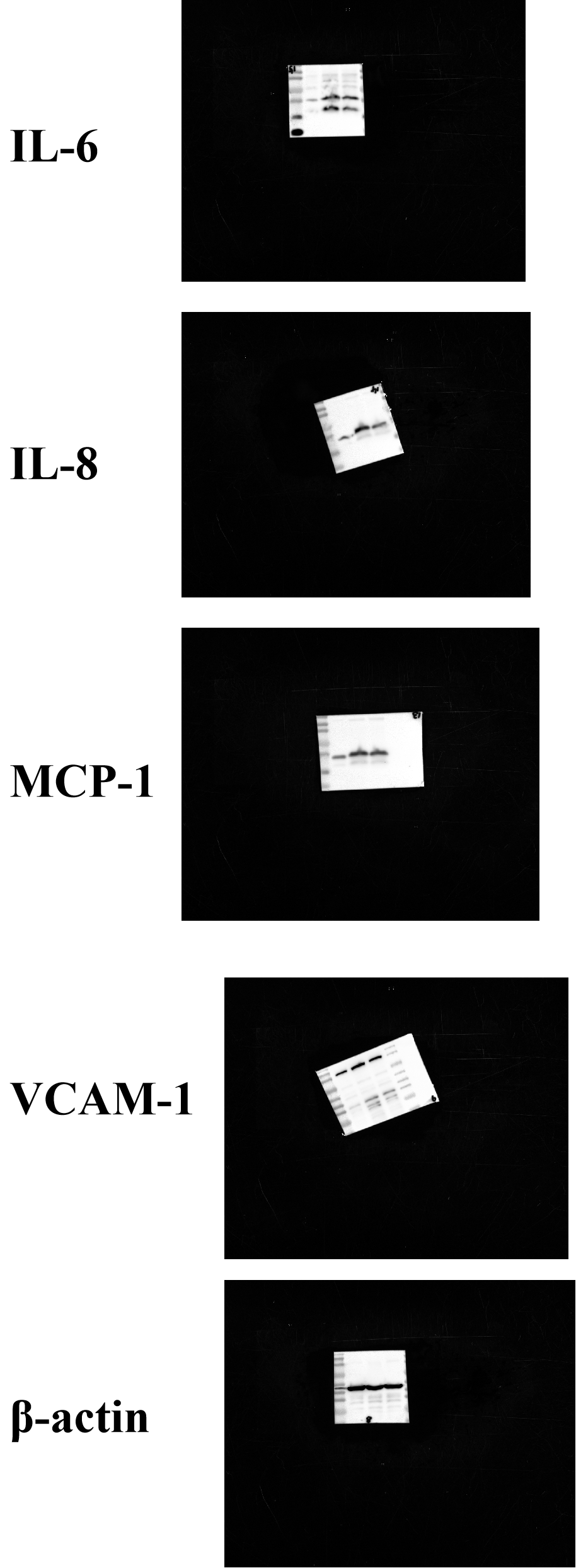


**Figure 4**


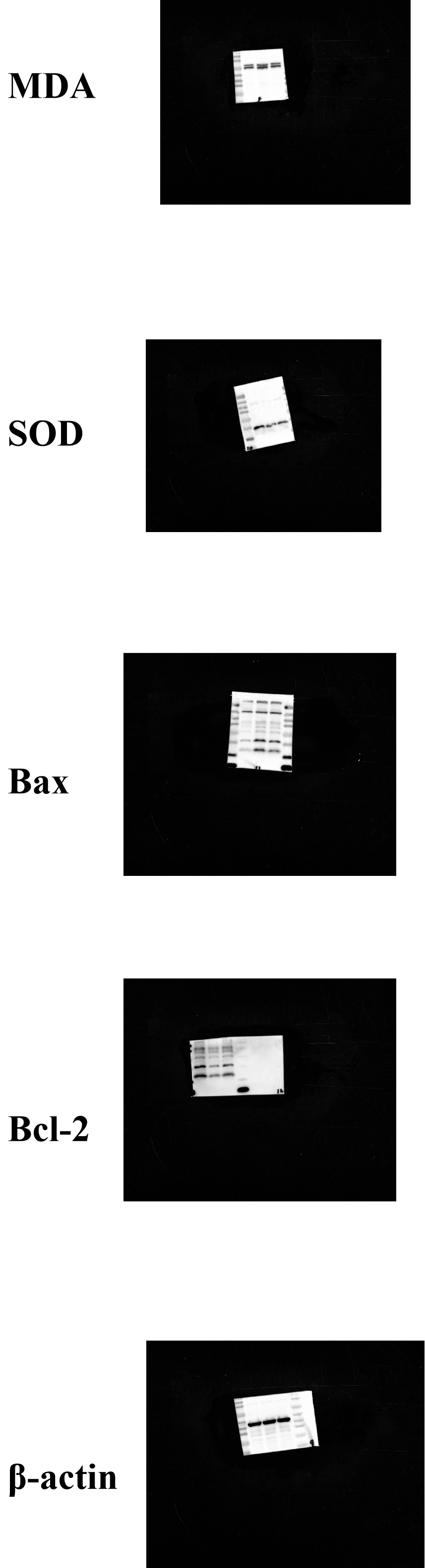


**Figure 5**


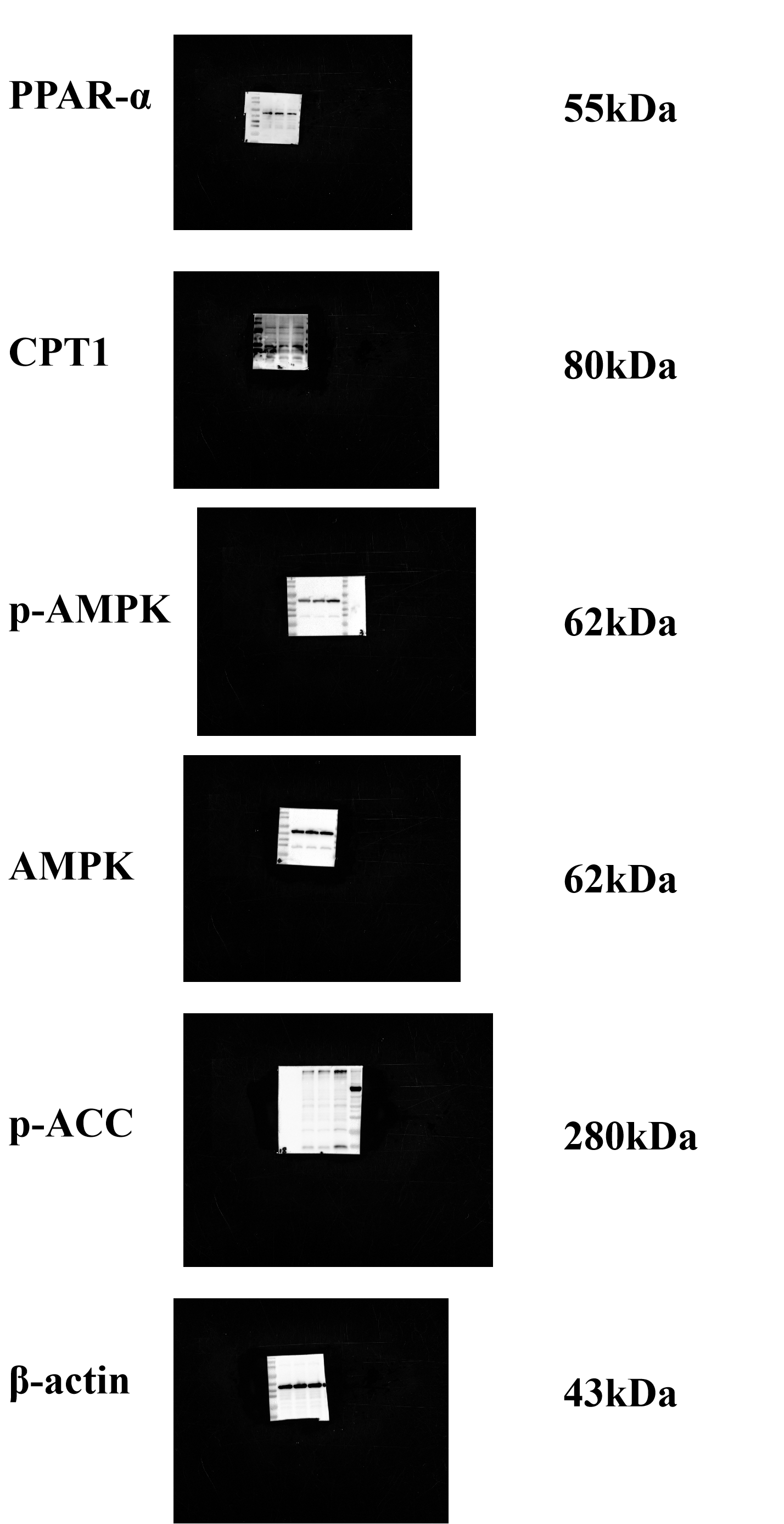


**Figure 6**


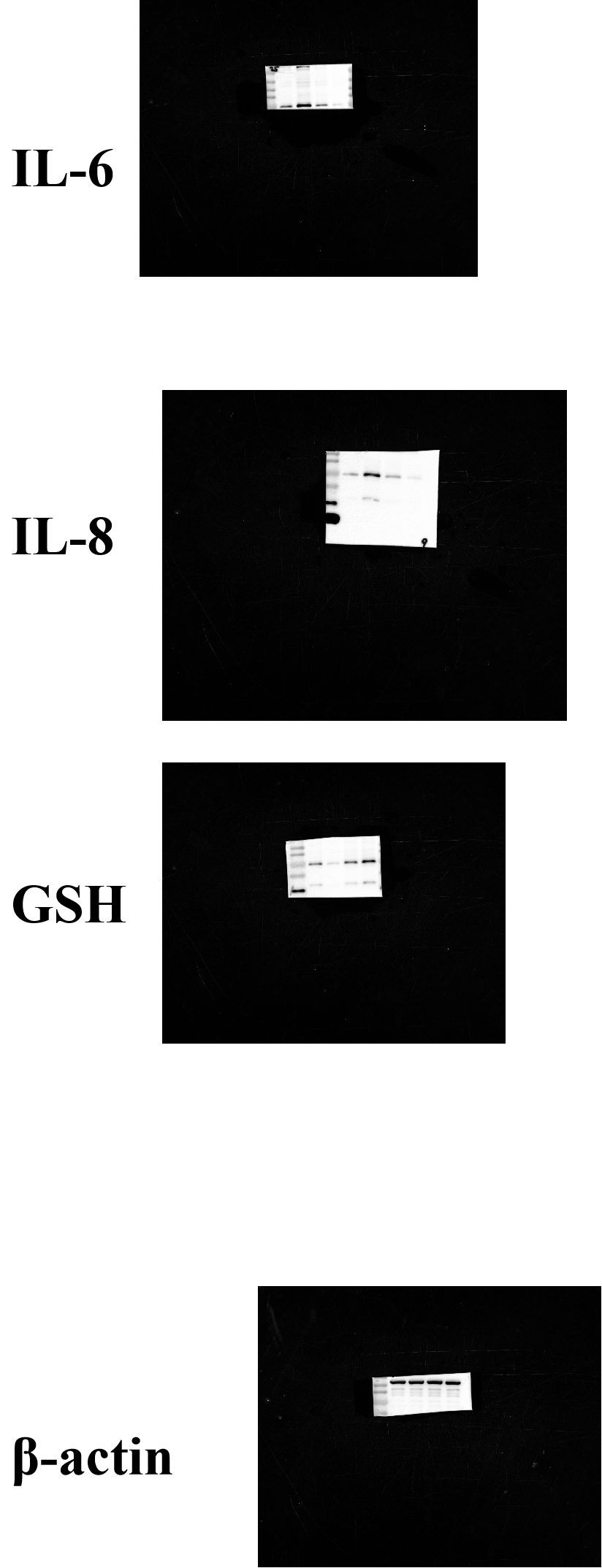


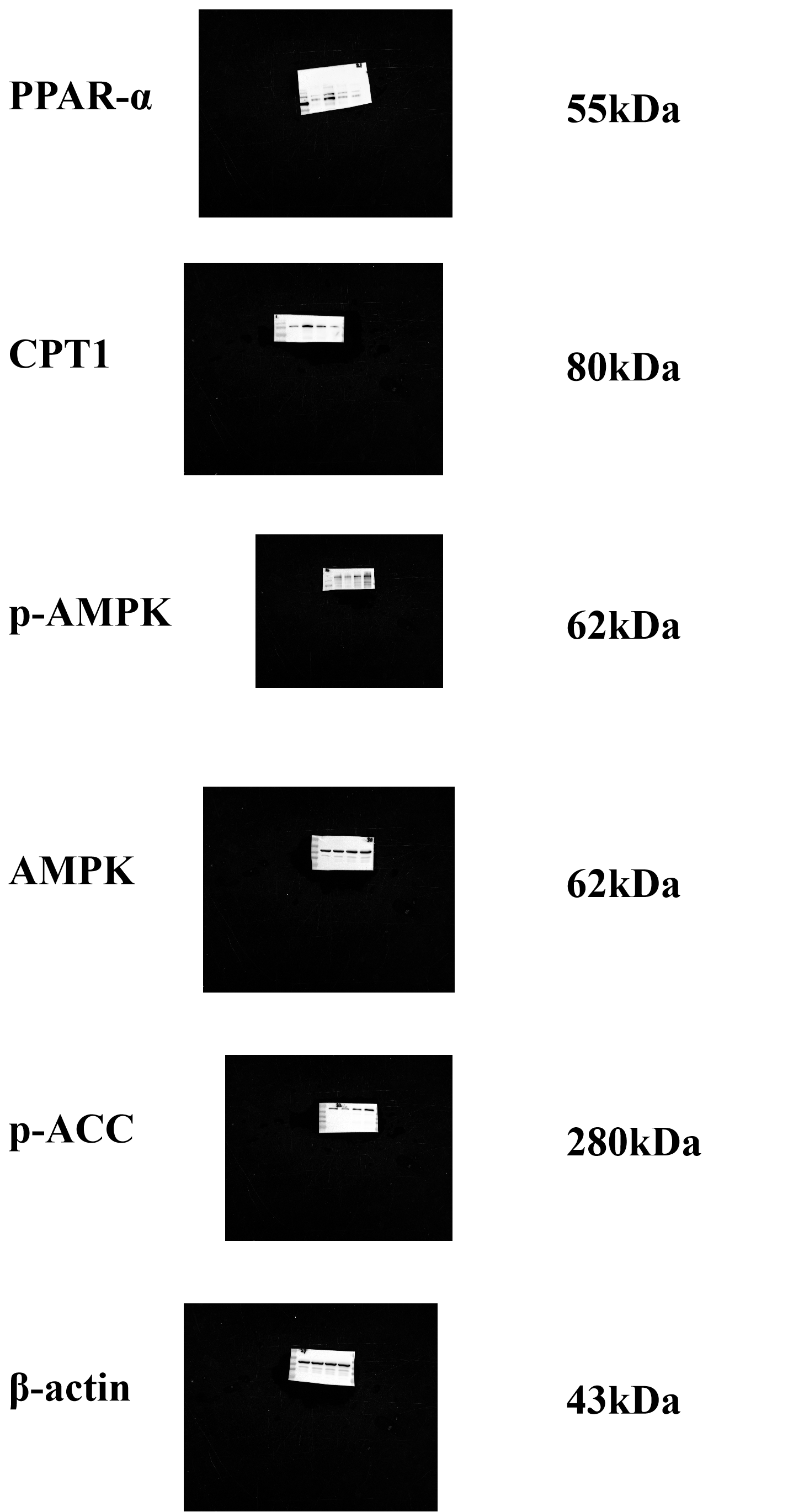


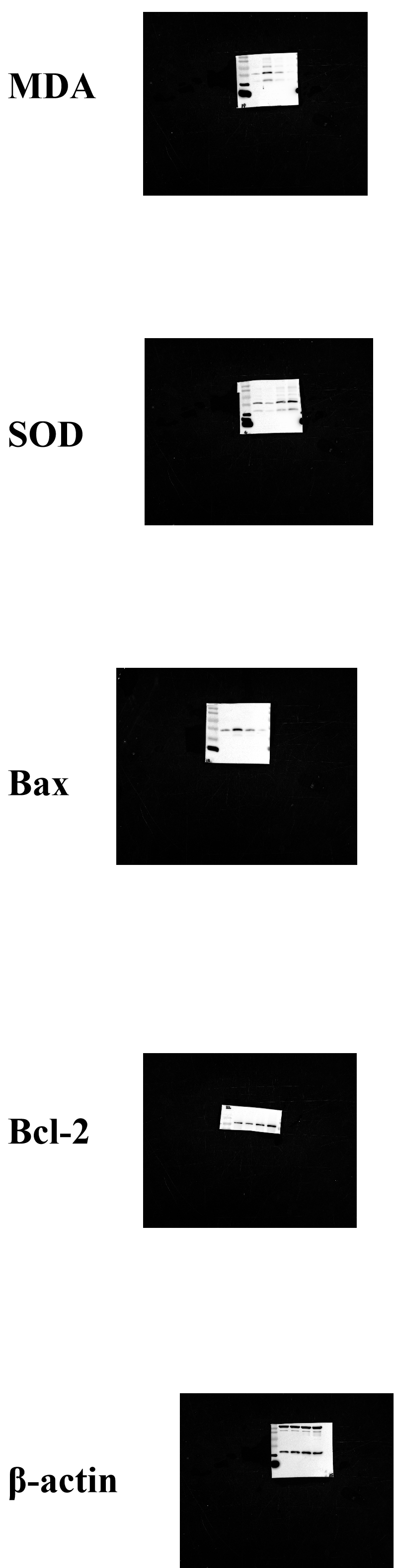


**Figure 7**


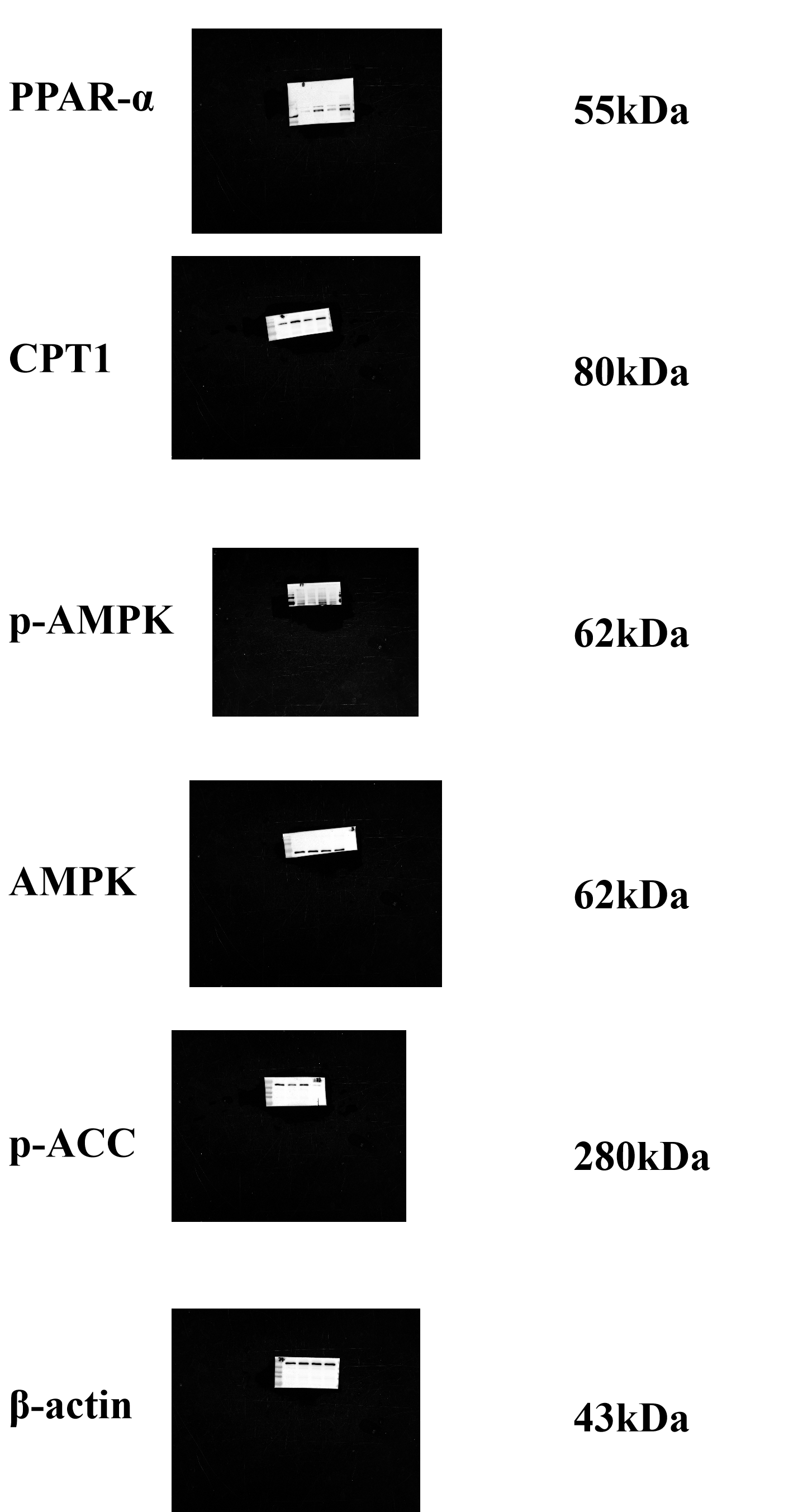


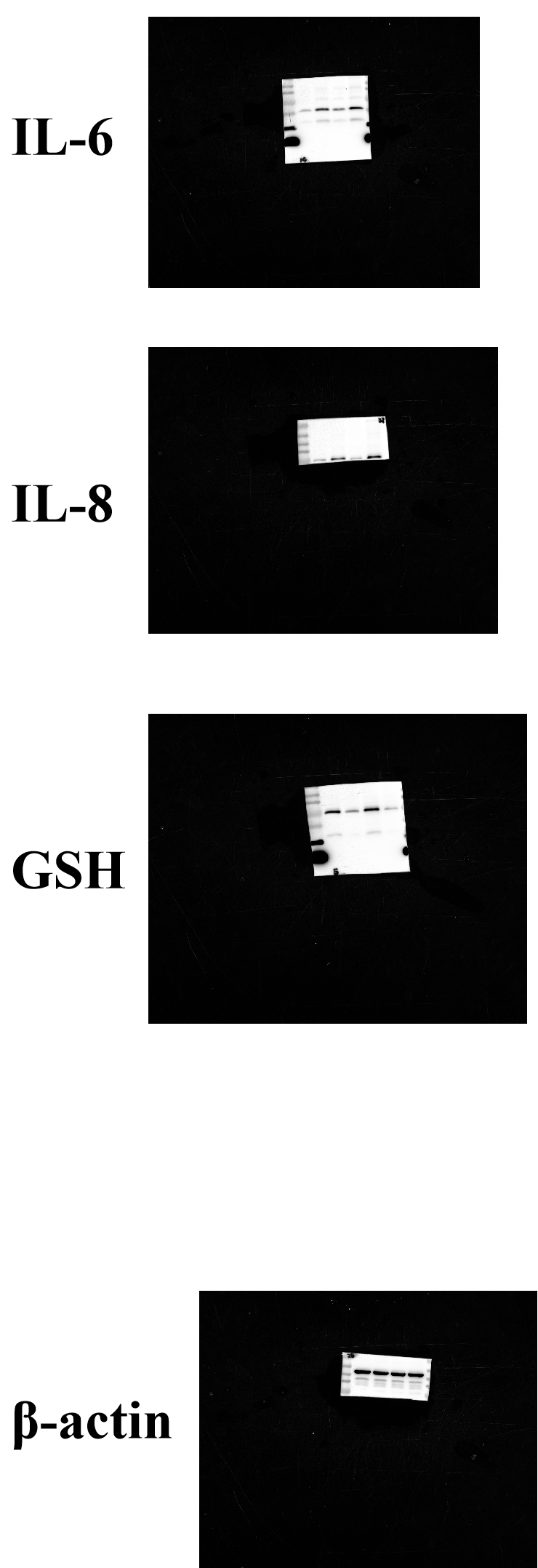


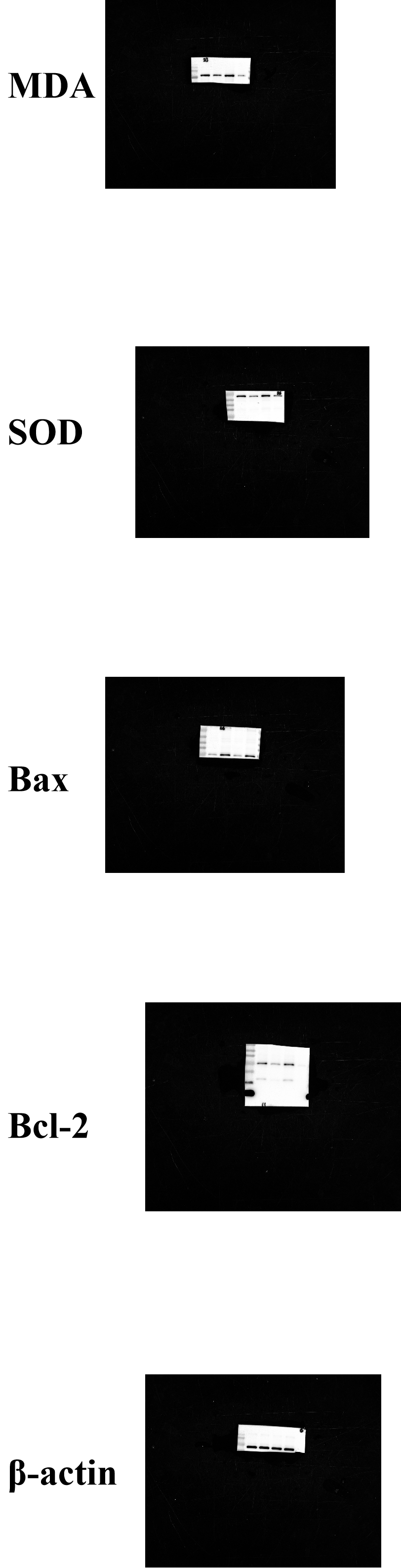

Supplement: Supplementary file 1 — Supplementary Material 1 [file 41598_2025_32157_MOESM1_ESM.doc]
